# Book Reviews

**Published:** 1897-09

**Authors:** 


DISEASES OF THE EAR, NOSE AND THROAT and their Accessory Cavities. A Condensed Text-book. By SETH SCOTT BISHOP, M. D., LL. D., Professor in the Chicago Post-Graduate Medical School and Hospital; Surgeon to the Illinois Charitable Eye and Ear Infirmary ; Consulting Surgeon to the Illinois Masonic Orphans' Home and to the Silver Cross Hospital of Joliet. Illustrated with 100 colored lithographs and 168 additional illustrations. Royal 8vo, pages xvi.—496. Philadelphia: The F. A. Davis Co., Publishers, 1914 and 1916 Cherry street.

This book is written in the interests of medical students and general practitioners in compliance with numerous requests from both of these important groups, and is not intended to replace exhaustive treatises on these subjects. Thus the author announces in his preface. That he has conscientiously attempted to fill this rôle cannot be gainsaid. The relationship between diseases of the ear, nose and throat have been recognised by the profession for a long time, while diseases of the eye constitute a separate field of specialism.

This work is well printed in plain, large-faced type and is quite copiously illustrated. Some of the engravings are original and a few of them printed in colors. We have been impressed with the author's account of the relationship between uric acid and hay fever—a factor in the causation of this disease which is often overlooked by specialists, we fear, or else given too little weight. Taken all in all we regard this treatise as a valuable contribution to the literature of the subjects with which it deals and believe it will be found a useful addition to the libraries of those for whom it is more especially written.

---

TRANSACTIONS OF THE SOUTHERN SURGICAL AND GYNECOLOGICAL ASSOCIATION. Volume IX. Ninth session held at Nashville, Tenn., November 10, 11 and 12, 1896. Edited by Dr. William Elias B. Davis, Secretary, Birmingham, Ala. Philadelphia: William J. Dornan, Printer. 1897.

Again this distinguished association sends to the professional world a volume of great value. Excellent as have been its predecessors, this book takes rank as one of the best volumes of transactions issued by this or any other special medical society. Many of the papers have been published in medical magazines, but in this volume only will be found the complete discussions which are strong, scientific and instructive. One of the most thoroughly enjoyable, scholarly and helpful papers in the book is by Dr. Lewis S. McMurtry, of Louisville, entitled, The evolution and perfection of aseptic surgical technique. It merits the attention of every practical surgeon. It would be interesting to analyse this paper as well as several others in the book, but just now it is impossible and perhaps unnecessary to do so, inasmuch as it is in the hands of every member of the association and every general as well as every gynecologic surgeon will be sure to obtain it.

---

A Clinical, Pathological and Experimental Study of Fracture of the Lower End of the Radius. With displacement of the Carpal Fragment Toward the Flexor or Anterior Surface of the Wrist. By John B. Roberts, A. M., M. D., Professor of Anatomy and Surgery in the Philadelphia Polyclinic ; Professor of Surgery in the Woman's Medical College of Pennsylvania, etc. Octavo, pp. 76. With 33 illustrations. Philadelphia : P. Blakiston, Son & Co., 1012 Walnut street. 1897.

The author of this brochure is an experienced anatomist and a forceful teacher of the subject, hence his writings not only command attention but a respectful examination. Perhaps there is no injury to bone structure more common than fracture of the lower end of the radius, with which the name of Colles has become almost classically associated. It, therefore, becomes every surgeon and especially every general practitioner in the country to carefully examine this monograph and to ponder its valuable teachings.

---

Fourteenth Report of the State Board of Health of the State of New Hampshire from July 1, 1895, to November 1, 1896. Irving A. Watson, M. D., Secretary. Concord : Edward N. Pearson, Public Printer. 1897.

The state of New Hampshire is fortunate in having an experienced sanitarian at the head of its health department in the person of Dr. Irving A. Watson, who acts as secretary of its board of health. The volume before us contains much of interest relating to questions of public health and vital statistics and may be examined with profit by all sanitarians.

---

Flint's Medical and Surgical Directory of the United States and Canada. Issued annually. 1897. Compiled by A. L. Chatterton. New York : J. B. Flint & Co., 104 Fulton street. 1897.

Medical directories are growing more numerous year by year ; and some of them are not as accurate as we could wish. The one before us, however, is perhaps as nearly so as it would be possible to make it considering the difficulties to be overcome. It has one decided advantage—namely, size and conciseness. It would be a good plan for compilers of directories to classify the names by counties, instead of arranging them alphabetically by cities, villages and hamlets. It is proposed to issue this directory annually, which commends it to favor, since errors can be corrected with greater promptitude than in biennial or triennial directories. Another advantage is, that Canada is embraced in the territory covered by this work.

---

LECTURES ON THE TREATMENT OF FIBROID TUMORS OF THE UTERUS, MEDICAL, ELECTRICAL AND SURGICAL. By FRANKLIN H. MARTIN, M. D., Professor of Gynecology Post-Graduate Medical School of Chicago; Surgeon Woman's Hospital of Chicago, etc. Chicago: The W. T. Keener Co. 1897.

This compilation of lectures by an experienced author is somewhat marred by the fact that it has evidently been published with a view, in large part, to advertise the Post-graduate Medical School of Chicago. It contains a number of illustrations that relate to that institution, which need not be regarded as necessary to elaborate the text. Nevertheless, it contains considerable valuable material that will be examined with interest by gynecologic surgeons. This remark has especial application to that part of the book devoted to the operative treatment of these tumors.

---

TRANSACTIONS OF THE MEDICAL ASSOCIATION OF CENTRAL NEW YORK. Twenty-ninth Annual Meeting, held at Rochester, N. Y., October 20, 1896. WILLIAM C. KRAUSS, M. D., editor. BUFFALO MEDICAL JOURNAL Print. 1897.

This association is fast acquiring a reputation for good work and it places its achievements upon record with skilful judgment. No association that fails to publish its scientific proceedings does itself justice. These volumes can be bound together in groups of five and this will make the association's work accessible in the library. The papers have already been published in the JOURNAL, hence need not be commented upon at this time. The secretary, Dr. William C. Krauss, is an experienced editor and has presented this little volume in excellent form.

---

TRANSACTIONS OF THE AMERICAN MICROSCOPICAL SOCIETY. Nineteenth Annual Meeting, held at Carnegie Library, Pittsburg, Pa., August 18, 19 and 20, 1896. WILLIAM C. KRAUSS, M. D., Secretary. Buffalo: A. T. Brown Printing House. 1897.

This handsome volume is a credit to the society whose proceedings it records. The editor has displayed skill and judgment in its preparation and the mechanical execution of the book is beyond criticism except in one particular—it deserves a more permanent cover. It is a work that ought to stimulate more frequent and thorough work with the microscope in medicine as well as in the other sciences. Some of the illustrations, too, are extremely beautiful. Taken by and large it is the most complete contribution to the literature of microscopy that the year has witnessed.

---

TWENTY-SECOND ANNUAL REPORT OF THE STATE BOARD OF HEALTH OF THE STATE OF MICHIGAN, for the Fiscal Year ending June 30, 1894. HENRY B. BAKER, M. D., Secretary. Lansing: Robert Smith & Co., State Printers and Binders. 1896.

The reports of this board are always valuable and constitute decided contributions to the literature of public sanitation. The board has an extensive library and publishes in each annual report a list of accessions thereto for the previous year. The secretary, Dr. Henry B. Baker, is an experienced sanitarian and his writings and compilations are often authoritatively quoted. This volume is uniform with its predecessors and is a welcome addition to the files.

SURGICAL HINTS FOR THE SURGEON AND GENERAL PRACTITIONER. By HOWARD LILIENTHAL, M. D., Assistant Attending Surgeon to Mt. Sinai Hospital, New York City. New York: International Journal of Surgery Company. 1897.

This small brochure contains personal observations and suggestions by the author which should prove useful in refreshing one's memory when he does not operate every day. It is handsomely printed and is of a convenient size for the pocket.

---

ANNUAL REPORT of the Supervising Surgeon-General of the Marine Hospital Service of the United States. For the fiscal year 1896. Washington: Government Printing Office. 1896.

This volume is especially interesting to quarantine officers and contains much valuable information upon cognate branches of public health service. The Marine Hospital Service has gained a reputation for the excellence of its reports and this one keeps full pace with its predecessors. Its value would, however, be considerably enhanced if it were bound in muslin. It is too large a book to trust to paper covers.

